# Colossal magnetoresistance in the multiple wave vector charge density wave regime of an antiferromagnetic Dirac semimetal

**DOI:** 10.1126/sciadv.adh0145

**Published:** 2023-10-13

**Authors:** Ratnadwip Singha, Kirstine J. Dalgaard, Dmitry Marchenko, Maxim Krivenkov, Emile D. L. Rienks, Milena Jovanovic, Samuel M. L. Teicher, Jiayi Hu, Tyger H. Salters, Jingjing Lin, Andrei Varykhalov, N. Phuan Ong, Leslie M. Schoop

**Affiliations:** ^1^ Department of Chemistry, Princeton University, Princeton, NJ 08544, USA.; ^2^ Helmholtz-Zentrum Berlin f‌ür Materialien und Energie, Elektronenspeicherring BESSY II, Albert-Einstein-Straße 15, 12489 Berlin, Germany.; ^3^ Materials Department and Materials Research Laboratory, University of California, Santa Barbara, Santa Barbara, CA. 93106, USA.; ^4^ Department of Physics, Princeton University, Princeton, NJ 08544, USA.

## Abstract

Colossal negative magnetoresistance is a well-known phenomenon, notably observed in hole-doped ferromagnetic manganites. It remains a major research topic due to its potential in technological applications. In contrast, topological semimetals show large but positive magnetoresistance, originated from the high-mobility charge carriers. Here, we show that in the highly electron-doped region, the Dirac semimetal CeSbTe demonstrates similar properties as the manganites. CeSb_0.11_Te_1.90_ hosts multiple charge density wave modulation vectors and has a complex magnetic phase diagram. We confirm that this compound is an antiferromagnetic Dirac semimetal. Despite having a metallic Fermi surface, the electronic transport properties are semiconductor-like and deviate from known theoretical models. An external magnetic field induces a semiconductor metal–like transition, which results in a colossal negative magnetoresistance. Moreover, signatures of the coupling between the charge density wave and a spin modulation are observed in resistivity. This spin modulation also produces a giant anomalous Hall response.

## Teaser

An antiferromagnetic Dirac semimetal shows colossal negative magnetoresistance.

## INTRODUCTION

Magnetoresistance (MR), i.e., a change (both increase and decrease) in the electrical resistivity of a material with application of a magnetic field, is an extensively studied phenomenon in condensed matter physics. Although known for decades, it remains relevant to this day due to the wide range of technological applications (*1*–*5*) and open questions about its origin (*6*–*9*) in a large variety of systems. Large positive MR can be generated solely by the Lorentz force in a clean material with long electron scattering lengths. This has been shown in high-purity bismuth and antimony crystals, where high-mobility carriers result in giant values of the positive orbital MR (*10*, *11*). In recent years, a very large positive MR has been reported in topological semimetals (TSMs) owing to a combination of relativistic quasiparticle excitations, high-mobility charge carriers, and highly anisotropic Fermi surface properties (*12*–*16*). On the other hand, negative MR has more complex origins and needs intricate theoretical models to explain. The most common negative MR appears because of magnetic order-disorder transition in magnetic materials. However, it only contributes to few tenths of percentage of change in resistivity. In contrast, depending on the observed features, types of material, and physical mechanisms involved, different terms have been introduced to categorize a large negative MR. For example, the giant MR (GMR) appears in ferromagnetic (FM)–nonmagnetic multilayer heterostructures, where a small magnetic field (about a few tenths of a tesla) results in electronic conduction between the spin-polarized layers and hence a sharp drop in resistivity (*1*, *2*, *17*, *18*). Colossal (negative) MR (CMR) is observed near the Curie temperature of hole-doped manganite perovskites, pyrochlores, and spinel compounds as a result of the transition from a paramagnetic insulating to an FM metallic state (*19*–*23*). However, the origin of CMR is different in each material family. Among these, manganites are the most explored, where, primarily, the double-exchange interaction (DEI) between mixed valence Mn ions leads to the metallic FM state (*24*–*26*). Because of the strong dependence of resistivity in a rather small magnetic field, both GMR and CMR materials attracted extensive interests in spintronics and magnetic memory applications (*1*–*5*). It is worth mentioning that in a special experimental configuration (collinear electric and magnetic fields), a weak negative MR can also be obtained in TSMs because of the relativistic chiral anomaly (*27*, *28*).

TSMs host linearly dispersing bands in their electronic band structure that are protected by crystallographic symmetries along with lattice inversion and/or time-reversal symmetry (*29*–*32*). Hence, they provide an opportunity to study the relativistic particle dynamics in low-energy systems. Through first-principles calculations, a large number of TSMs have been identified over the past few years (*33*–*36*). Square-net materials are candidates that show probably the cleanest signatures of nontrivial topological bands (*37*–*43*). These compounds have a two-dimensional square-net atomic motif in the crystal structure, which produces isolated Dirac cones at the Fermi energy (*E*_F_) in the electronic band structure (*44*) with the largest reported energy range of linear dispersion (*37*). LnSbTe (Ln = lanthanides), which contain magnetic rare-earth elements, is a subgroup of the square-net family, and they make up one of the rare examples of time-reversal symmetry–broken magnetic Dirac semimetals (*40*–*43*, *45*). Furthermore, by changing the number of electrons per atom at the Sb square-net via chemical substitution, structural distortions and charge density wave (CDW) can be induced in LnSbTe (*46*–*49*). While electron filling moves the Fermi energy, thus offering access to other bands in the electronic transport experiments, CDWs open gaps at the Fermi surface and yield additional quantum states. It has been shown that CDWs clean the band structure, create an ideal nonsymmorphic Dirac semimetal state in electron doped GdSbTe (*50*), and lead to rich magnetism and a potential skyrmionic phase (*51*).

**Fig. 1.**
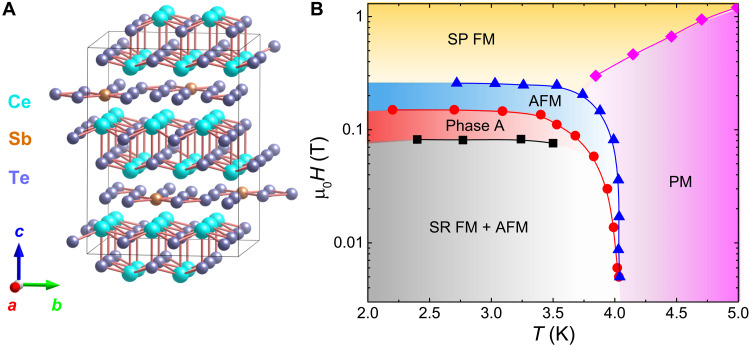
Crystal structure and phase diagram of CeSb_0.11_Te_1.90_. (**A**) Crystal structure of CeSb_0.11_Te_1.90_ reconstructed from data in (*48*). (**B**) Magnetic phase diagram for CeSb_0.11_Te_1.90_. The data points are extracted by digitizing the phase diagram presented in (*48*). The phase boundaries separate the AFM, short-range FM (SR FM), spin-polarized FM (SP FM), and paramagnetic (PM) states. Phase A represents a field-induced spin-modulated state that leads to a devil’s staircase behavior in magnetization.

Here, we focus on the anti-FM (AFM) Dirac semimetal CeSbTe (*40*). With application of an external magnetic field, the band structure of CeSbTe can be tuned to realize Weyl and higher-order topological states (*40*). Recently, by substituting Sb with Te on the square-net site, the evolution of the CDW at room temperature has been investigated in CeSb*_x_*Te_2−*x*−δ_ (δ is the vacancy concentration in the crystal) (*48*). A CDW appears at around *x *< 0.79 accompanied by a structural distortion from the tetragonal to an orthorhombic phase. The associated modulation wave vector (**q**) changes continuously as a function of *x*. At the highest electron filling range 0.10 ≤ *x *< 0.34, a complex CDW ordering is observed, represented by multiple **q** vectors. The corresponding crystal structure is shown in Fig. 1A. The CDW also modifies the AFM ground state. Especially in the region of multiple **q** vectors, a magnetic field–induced “devil’s staircase” ordering is reported in the magnetization (Fig. 1B) (*48*). A series of fractionally quantized magnetization plateaus originate from the coupling between the CDW and a spin modulation along the *c* axis. First-principles calculations suggest that a rigid band model can be assumed where the electron filling moves *E*_F_ and the CDW gaps out a number of band crossings. At *x* = 0.11, several Dirac nodes persist at or near *E*_F_ (*48*). In this report, we combine angle-resolved photoemission spectroscopy (ARPES) and electronic transport measurements to unveil the interplay between the CDW, spin modulation, and topological states in the multiple **q** vector regime of CeSb*_x_*Te_2−*x*−δ_.

## RESULTS

### ARPES results and topological states

**Fig. 2.**
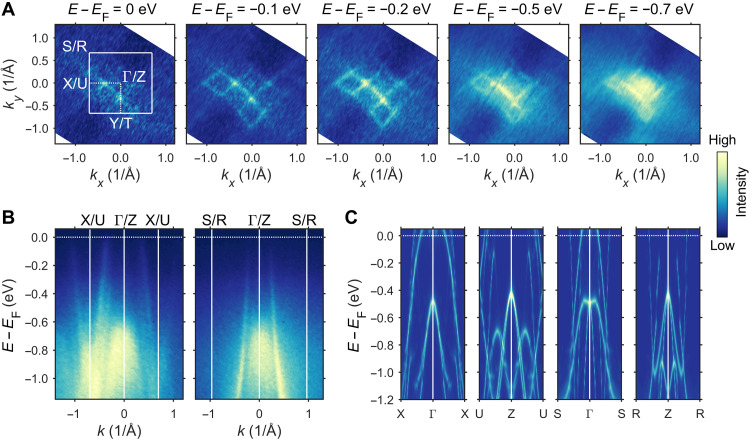
ARPES spectra for CeSb_0.11_Te_1.90_ at 1.5 K measured with a photon energy of 110 eV. (**A**) Constant energy contours at different energy (*E*) values from the Fermi energy (*E*_F_) to *E*_F _− 0.7 eV. (**B**) Experimental and (**C**) theoretical electronic band dispersion along the high symmetry directions *X*(*U*)-Γ(*Z*)-*X*(*U*) and *S*(*R*)-Γ(*Z*)-*S*(*R*).

In Fig. 2, we present the results of the ARPES measurements, measured with a photon energy of 110 eV (see fig. S1 for an additional photon energy). The experiments were performed at 1.5 K, which is below the reported AFM Néel temperature (*T*_N_) of 4.27 K for CeSb_0.11_Te_1.90_ (*48*). In Fig. 2A, we show the constant energy contours at different energy (*E*) values from *E*_F_ to *E* = *E*_F_ − 0.7 eV. Similar to CeSbTe (*40*), a diamond-shaped Fermi surface is observed. Unlike the parent compound, Fermi surface nesting and corresponding energy gap opening are expected in CeSb*_x_*Te_2−*x*−δ_ for *x *< 0.79 due to the presence of the CDW. Despite the strong CDW modulation observed in CeSb_0.11_Te_1.90_ in single crystal x-ray diffraction (*48*), the band structure remains rather metallic. The previously reported theoretical band structure for CeSb_0.11_Te_1.90_ showed that several Dirac cones cross the Fermi energy with the nodes residing above *E*_F_ (*48*). The positions of these Dirac nodes are also highlighted in fig. S3A. Along the Γ-*X* and Γ-*S* directions, the Dirac points are near *E*_F_. In Fig. 2 (B and C), the measured band dispersions and the calculated band structure, respectively, are plotted along the high-symmetry directions *X*-Γ-*X* and *S*-Γ-*S*. For both directions, clean, linearly dispersing bands are observed, which agree well with the theoretical results. These bands can be clearly resolved down to *E* = *E*_F _− 1.0 eV, therefore confirming the robustness of the Dirac semimetal state in CeSb_0.11_Te_1.90_. To provide an easier comparison, we have overlaid the calculated band structure with the ARPES spectra in fig. S2. The ARPES spectra measured with photon energy of 70 eV at ∼30 K (fig. S1) also shows similar features, thus indicating that the AFM ordering does not modify the topological electronic state. To clearly show the effect of the CDW, in fig. S3, we have compared the electronic band structure of CeSb_0.11_Te_1.90_ and calculated using the CDW-modulated and a hypothetical unmodulated crystal structure. It is evident that the CDW reduces the spectral weight of several bands near *E*_F_, thus indicating a partial gap opening at few parts of the Fermi surface, which is expected in a three-dimensional system. We note that the intensity of the ARPES spectrum diminishes as we approach *E*_F_, thus making it difficult to identify the points at which the bands cross the Fermi energy. In fig. S4, we show the ARPES results obtained from the energy distribution curves normalized by the sum of energy distribution curves over all *k_x_* and *k_y_* values. This method enhances the intensity of the weak features in the ARPES spectrum. From the normalized data for both photon energies, we can clearly see the bands crossing the Fermi level.

### Semiconducting properties and semiconductor metal–like transition

**Fig. 3.**
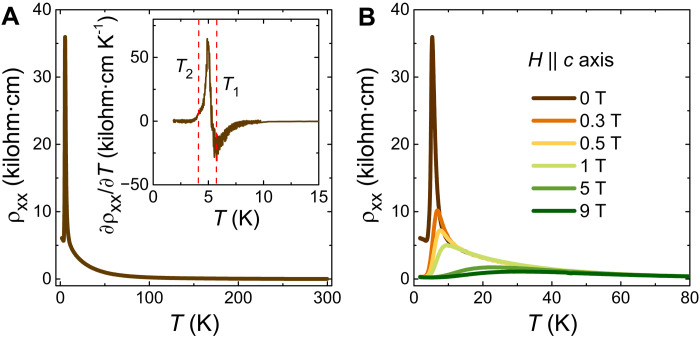
Temperature-dependent electronic transport properties of CeSb_0.11_Te_1.90_. (**A**) Temperature (*T*) dependence of the resistivity (ρ*_xx_*). Inset shows the first-order derivative of ρ*_xx_*(*T*), revealing two transition temperatures (dashed vertical lines). (**B**) ρ*_xx_*(*T*) curves when an external magnetic field is applied along the crystallographic *c* axis.

Despite the presence of a Fermi surface in ARPES and a finite density of states at *E*_F_ in density functional theory calculations (fig. S5), the resistivity (ρ*_xx_*) for CeSb_0.11_Te_1.90_ shows a semiconductor-like temperature (*T*) dependence (Fig. 3A) when the current is applied along the *b* axis. From 300 K, ρ*_xx_* increases monotonically with decreasing temperature, followed by a sharp peak and sudden drop in the low-temperature region. The first-order derivative of ρ*_xx_*(*T*) in the inset of Fig. 3A reveals two transition temperatures, *T*_1_ ∼ 5.8 K and *T*_2_ ∼ 4 K. Although the change in the slope near *T*_2_ is subtle, a prominent signature is observed for another crystal (sample 2), from the same batch, as plotted in fig. S6. At this electron filling for CeSb*_x_*Te_2−*x*−δ_, a short-range FM ordering is reported to coexist with the AFM ground state (*48*). The ordering temperature (*T*_C_) of this FM state is just below *T*_N_. By comparing the resistivity with the previously reported magnetization data (*48*), we conclude that *T*_1_ corresponds to the AFM transition, whereas *T*_2_ represents *T*_C_. Both of these transition temperatures are a bit higher as compared to those obtained from magnetization measurements. Upon the application of an external magnetic field (*H*) along the crystallographic *c* axis, the ρ*_xx_*(*T*) curve undergoes a marked change (Fig. 3B). With a field of just 0.3 T, the resistivity drops to one-third of the maximum value at *T*_1_ and the peak shifts to higher temperature. This trend continues with increasing field strength and a semiconductor metal–like transition becomes apparent in the low-temperature region. Even at the highest field of 9 T, a broad maximum persists at around 30 K. The observed features suggest that a very high magnetic field might be needed to completely suppress the semiconductor-like behavior. Similar properties are also obtained for sample 2 (fig. S6). From the reported magnetization data, we know that for CeSb_0.11_Te_1.90_, a fully spin-polarized state can be achieved at a small field of ∼0.2 T (*48*). Therefore, magnetic ordering alone cannot explain the low-temperature behavior of ρ*_xx_*.

To understand the overall nature of the ρ*_xx_*(*T*) curve, let us discuss the usual possibilities. The presence of the Kondo effect from the *f*-electrons of cerium could lead to a semiconducting behavior. However, in Kondo materials, ρ*_xx_* decreases with decreasing temperature, followed by a region of saturation and then an enhancement at low temperature (*52*). In CeSb_0.11_Te_1.90_, in contrast, above *T*_N_, ρ*_xx_* never decreases with decreasing temperature. Moreover, in Ce-based heavy fermionic systems, ARPES measurements with a photon energy above ∼100 eV revealed prominent coherent Kondo flat bands at the Fermi level (*53*). From our ARPES results at 110 eV (Fig. 2B), we can confirm that these bands are not present in CeSb_0.11_Te_1.90_ near *E*_F_. Following the activated transport model of an intrinsic semiconductor, we have plotted lnρ*_xx_* as a function of 1/*T* in fig. S7A. It is evident that the slope of this curve changes continuously with temperature. Hence, a unique value of any possible CDW-induced energy gap cannot be obtained. We note that the lnρ*_xx_*(1/*T*) curves for all field strengths collapse on the zero-field data above ∼100 K (fig. S7A). From the slope of two approximately linear regions, we estimate the energy gap to be ∼2.4 and ∼0.85 meV with a smooth cross-over between them. Another possible origin of this semiconductor-like behavior can be localization effects due to the intrinsic lattice vacancies (δ) in the single crystals (*54*). A weak localization effect emerges from quantum interference between scattering paths of carriers at very low temperature. However, in the case of CeSb_0.11_Te_1.90_, the semiconductor-like transport is very robust up to room temperature. On the other hand, Anderson localization occurs in strongly disordered electron systems at low temperature and gets suppressed under a weak magnetic field. While the resistivity in CeSb_0.11_Te_1.90_ indeed decreases notably with a small applied field (<1 T) around and below *T*_N_ (probably because of the spin-scattering), the change becomes gradual above *T*_N_, and ρ*_xx_* is found to be insensitive to the magnetic field above ∼100 K. Instead, the temperature dependence of the zero-field resistivity is better described by a ρ*_xx_* = *AT*^−*n*^-type power law, where *A* and *n* are arbitrary parameters. Two distinct regions have been observed with the exponent *n* ∼ 6 for 240 ≤ *T* ≤ 300 K and *n* ∼ 2 for 60 ≤* T *≤ 150 K (fig. S7B). This unusual power law behavior cannot be explained by the conventional scattering models and warranted further theoretical/microscopic investigations. We note that an unconventional power law–dependent resistivity has also been reported for the nonsymmorphic Dirac semimetal GdSb_0.46_Te_1.48_, possibly as a result of nonsymmorphic Dirac fermions in presence of CDW together with the lattice disorder (*50*). However, in the case of GdSb_0.46_Te_1.48_, the obtained exponent *n* ∼ 0.3.

### Colossal MR

**Fig. 4.**
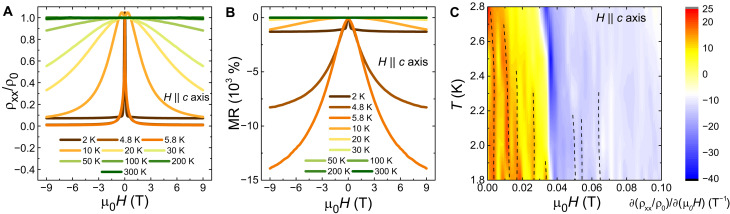
Magnetic field dependent electronic transport properties of CeSb_0.11_Te_1.90_. (**A**) Magnetic field (*H*) dependence of the normalized resistivity (ρ*_xx_*/ρ_0_) at different temperatures with field applied along the *c* axis. (**B**) Field dependence of the MR at different temperatures. (**C**) Phase diagram constructed from the first-order derivative of ρ*_xx_*/ρ_0_ with respect to the magnetic field. The black dashed lines highlight the evolution of the transitions in the ρ*_xx_*/ρ_0_(*H*) curves with temperature.

It is clear that the magnetic field markedly modulates the transport properties of CeSb_0.11_Te_1.90_. In Fig. 4A, we show the field dependence of the normalized resistivity (ρ*_xx_*/ρ_0_) at different temperatures, where ρ_0_ is the zero-field resistivity. For both ρ*_xx_* and the Hall resistivity (ρ*_yx_*), a prominent hysteresis is observed between field sweep-up and sweep-down curves. This is a known problem with magnetic materials and requires special attention to avoid any spurious signal during symmetrization or antisymmetrization of the MR and Hall data, respectively. Therefore, we have adopted a modified symmetrization/antisymmetrization technique (*55*) to circumvent this issue as described in the Supplementary Materials. For *T* ≤ *T*_N_, ρ*_xx_*/ρ_0_ drops sharply in the low field region 0 < μ_0_*H *≤ 1 T, followed by almost saturation-like behavior at higher field strengths. This has some similarities with GMR systems, which are excellent candidates for spin-valve applications (*1*, *2*). We note that the decrease in resistivity is particularly steep near *T*_N_, whereas it fades off as we move up/down in temperature. This is consistent with the suppression of the spin scattering by an external field in magnetic materials. For *T* > *T*_N_, the change in resistivity becomes much more gradual. It is worth mentioning that at low temperatures, ρ*_xx_* shows a slight increment in the very narrow field range 0 < μ_0_*H *≤ 0.03 T (fig. S8A), which could be due to a weak antilocalization effect.

As evident from Fig. 4A, similar to manganites (*19*–*21*), the magnetic field–induced semiconductor metal–like transition produces a very large negative MR in CeSb_0.11_Te_1.90_. The maximum value of the negative MR, calculated by usual convention, is always restricted to −100%. Hence, here, we use the definition $\mathrm{MR}=\frac{\rho_{xx}(H)-\rho_{0}}{\rho_{xx}(H)}\times 100\%$, as commonly used for the manganites (*21*). At 2 K with a field of 9 T, the MR is found to be −1300%, which increases to −14,000% at *T*_N_ (Fig. 4B). To the best of our knowledge, such a large CMR has not been observed in TSMs so far and is only one order of magnitude smaller than the “thousandfold change in resistivity” in the heat-treated La_0.67_Ca_0.33_MnO*_x_* epitaxial film (*21*). Sample 2, which is a crystal from the same batch and may have an arbitrarily different lattice vacancy, also shows similar behavior with maximum MR = −530% (fig. S9A). Furthermore, Murakawa *et al.* (*56*) recently reported a field-induced sharp decrease in resistivity at the doping range *x* = 0.17 for CeSb*_x_*Te_2−*x*−δ_. Therefore, the observed CMR is an intrinsic property of CeSb*_x_*Te_2−*x*−δ_ in the heavily electron-doped, multiple **q** vector regime. Although the MR value diminishes with increasing temperature, it remains noticeable (−14%) even at 50 K, which is way above *T*_N_. The MR also has a substantial anisotropy with respect to the direction of the applied magnetic field. As illustrated in fig. S10, keeping the current direction unaltered, rotation of the magnetic field in the *ac* plane generates a twofold symmetric pattern of the resistivity with ρ*_xx_* decreasing much more slowly for fields along the *a* axis. From the obtained results, we estimate an anisotropy ratio $\left(\rho_{xx}^{H\|a}/\rho_{xx}^{H\|c}\right)$ of ∼2.6 at 5.4 K and a magnetic field of 9 T. The large anisotropy ratio is also a confirmation of the layered crystal structure and corresponding quasi–two-dimensional nature of the Fermi surface in CeSb_0.11_Te_1.90_.

We note that the observed field–induced semiconductor metal–like transition and the overall magnetotransport properties have similarities with different types of CMR materials rather than TSMs. Among these CMR systems, in doped manganites, the MR is primarily originated from the DEI between mixed valence Mn ions and or the charge/orbital ordering (*24*–*26*). However, DEI alone cannot explain the resistivity behavior in manganites (*8*). Relatively recently, the randomly distributed disorder (quenched disorder) in a doped system has been identified as the major factor in influencing the length and time scale at the critical electronic phase separation region between the charge/orbital order and metallic ferromagnetism in manganites (*57*–*59*). This quenched disorder is thus suggested to play an important role in generating the CMR. In the case of CeSb_0.11_Te_1.90_, heat capacity and magnetic measurements suggest that there is no substantial valence fluctuation in the Ce ions (*48*). The quenched disorder in the highly doped crystals is therefore likely to be a possible mechanism responsible for the CMR in this material. Nevertheless, further experimental/theoretical insights would be needed to confirm its origin. The observed electronic transport behavior in CeSb_0.11_Te_1.90_ is similar to the AFM insulator Eu_5_In_2_Sb_6_ (*60*). Analogous to the Dirac semimetal CeSbTe, Eu_5_In_2_Sb_6_ is a nonsymmorphic Zintl phase, albeit with a bandgap. From theoretical calculations, an axion insulator state is proposed in its AFM state (*60*), although subsequent calculations found a trivial ground state (*61*). The field-induced metal-insulator transition and a CMR of ∼10^6^% are shown to be due to the formation of magnetic polarons, which also results in a deviation of magnetization from the Curie-Weiss behavior starting well above the Neél temperature (*60*). Among pyrochlore iridates, the CMR in Nd_2_Ir_2_O_7_ reaches ∼10^4^% at 10 T when the magnetic field is applied along the (001) axis (*62*). Nd_2_Ir_2_O_7_ has a zero-field semimetallic paramagnetic state, which is followed by an insulating state at the magnetic ordering temperature of ∼32 K. The insulating state is easily affected by an external magnetic field, possibly due to the proximity to a tricritical point (*62*). The strong correlation of the Ir electrons as well as the Nd_4f_ and Ir_5d_ spin configurations are shown to be important to explain the observed properties (*62*, *63*). A more recent example of a CMR compound is the ferrimagnetic insulator Mn_3_Si_2_Te_6_. This compound shows a CMR of ∼10^9^% if the field is applied along the magnetic hard axis, i.e., by avoiding fully spin-polarized state (*64*). The anisotropic CMR is predicted to be caused by the lifting of the topological nodal-line degeneracy of the spin-polarized bands depending on the spin orientation (*65*). Other notable examples include Eu-based insulators EuMnSb_2_ (*66*) and EuTe_2_ (*67*), both of which show a CMR value of 10^5^ to 10^7^% below the magnetic ordering temperature. In these materials, a magnetic field–induced change in the electronic band structure is proposed.

### Coupling of the CDW and spin-modulation

For *H*‖*c* axis, below *T*_N_, the ρ*_xx_*/ρ_0_ curves show a series of weak transitions within the low-field range of 0 < μ_0_*H *≤ 0.2 T (fig. S8A). These transitions become readily distinguishable as we take the first-order derivative of ρ*_xx_*/ρ_0_ with respect to the magnetic field (fig. S8B). In Fig. 4C, we have constructed a phase diagram by plotting ∂(ρ*_xx_*/ρ_0_)/∂(μ_0_*H*) as a function of temperature and field. As highlighted by the black dashed lines, the temperature evolution of these transitions can be clearly identified. Similar weak transitions are also observed for sample 2 (fig. S9B). For CeSb*_x_*Te_2−*x*−δ_, only within 0.10 ≤ *x *< 0.34, CDW modulation wave vectors extend along the crystallographic *c* axis (*48*). This CDW gets coupled with the spin modulation, which is created by the alternating spin up/down Ce^3+^ layers along the *c* axis in the AFM state. With an external magnetic field, the wavelength of the spin modulation can be tuned, and as a result, these coupled excitations can be driven through successive phase-locked and nonphase-locked states (*48*). Therefore, physical properties involving electronic charge density or spins should both show signatures of these coupled states. In CeSb_0.11_Te_1.90_, similar to the fractionally quantized plateaus in magnetization (*48*), the resistivity also shows a string of these transitions. We note that as in magnetization, these transitions disappear in the fully spin-polarized state at ∼0.2 T or in the paramagnetic region above *T*_N_ due to the absence of any spin modulation.

### Anomalous Hall effect

**Fig. 5.**
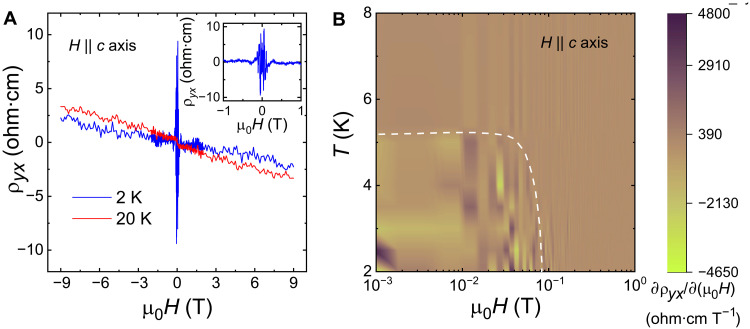
Hall measurements for CeSb_0.11_Te_1.90_. (**A**) Magnetic field dependence of the Hall resistivity (ρ*_yx_*) below and above the Néel temperature. The inset shows the low-field region of ρ*_yx_* at 2 K. (**B**) Phase diagram obtained from the first-order derivative of ρ*_yx_* with respect to the magnetic field. The white dashed line represents the phase boundary for the AHE.

In Fig. 5A, we have plotted the Hall resistivity as a function of magnetic field at two representative temperatures, above and below *T*_N_. ρ*_yx_*(*H*) is approximately linear with a negative slope. From the slope in the high-field region at 2 K (fig. S12), we estimate electron-type carriers with a density of ∼1.5 × 10^15^ cm^−3^, which is comparable to intrinsic semiconductors and consistent with the electronic transport properties observed for CeSb_0.11_Te_1.90_. The mobility at 2 K is found to be ∼0.69 cm^2^ V^−1^ s^−1^. It is interesting that despite the presence of the steep, linearly dispersing bands near the Fermi energy, CeSb_0.11_Te_1.90_ has such a low carrier mobility. However, it is not unexpected as the scattering length of the carriers must be very short in the highly disordered crystals due to the lattice vacancy. As already discussed, this quenched disorder also plays an important role in generating the CMR in this material. The ρ*_yx_*(*H*) curve at 2 K shows a prominent discontinuity near the zero-field limit as evident from the inset of Fig. 5A. We confirm that this anomalous Hall effect (AHE) is not a spurious signal coming from the hysteresis during the antisymmetrization of the experimental data. In fig. S11 (B and C), we have plotted ρ*_yx_*(*H*), calculated from the conventional method when the hysteresis is neglected. Although it also shows an anomalous behavior, the nature and amplitude are substantially different. In Fig. 5B, a phase diagram is constructed by plotting ∂ρ*_yx_*/∂(μ_0_*H*) as a function of temperature and magnetic field. A phase boundary (indicated by the white dashed line) is obtained, which separates the AHE region. We note that this phase boundary almost coincides with the AFM state in magnetic phase diagram. Hence, an AHE is not expected for a purely AFM ground state with vanishingly small magnetic moment, it could be originated from the collective spin excitation in the low-field limit or the short-range FM ordering. It is worth mentioning that because of the strong CMR in CeSb_0.11_Te_1.90_, the raw Hall data unavoidably contain a large longitudinal resistivity component. Furthermore, in this material, ρ*_xx_* is about four orders of magnitude higher than ρ*_yx_*. Hence, the extracted Hall data from the antisymmetrization technique is always noisy. However, we confirm that the observed anomalous Hall behavior is reproducible over multiple measurements on both samples 1 and 2.

We know that the Hall resistivity in a magnetic material follows the relation $\rho yx=\rho_{yx}^{O}+\rho_{yx}^{A}$, where $\rho_{yx}^{O}$ is the ordinary Hall resistivity and $\rho_{yx}^{A}$ represents the anomalous Hall component. $\rho_{yx}^{O}$ is proportional to the magnetic field and can be estimated by extrapolating the linear fitting of the high-field region of the ρ*_yx_*(*H*) data (fig. S12). For CeSb_0.11_Te_1.90_, we obtain an anomalous Hall resistivity of ∼8 ohm·cm at 2 K. This value in semiconducting CeSb_0.11_Te_1.90_ is giant in comparison to the metallic noncollinear/noncoplanar antiferromagnets where $\rho_{yx}^{A}$ is typically of the order of a few microhm-centimeter (*68*, *69*). However, because of the very large value of the longitudinal resistivity in CeSb_0.11_Te_1.90_, the anomalous Hall conductivity ($\sigma_{xy}^{A}\approx \frac{\rho_{yx}^{A}}{\rho_{xx}^{2}}$) is calculated to be rather small ∼10 μS cm^−1^. In metallic noncollinear/noncoplanar antiferromagnets, on the other hand, small longitudinal resistivity results in $\sigma_{xy}^{A}∼10$ to 200 S cm^−1^ (*70*).

## DISCUSSION

Beside the fundamental physics of massless relativistic particles, the unusual electronic transport properties drove the interest toward topological materials. Different mechanisms were proposed to decipher the large positive MR in these systems (*71*). However, high-mobility charge carriers appear to be the most plausible explanation. On the other hand, doped manganites belong to the realm of strongly correlated systems, which demonstrate metal-insulator transitions and CMR. Realization of these features in a topological material is unexpected. The presence of the complex CDW state and its coupling with the spin modulation in this high-electron filling region of AFM CeSb*_x_*Te_2−*x*−δ_ makes it an even more interesting system. Although the material is semiconducting, it exhibits a Fermi surface; thus, the CDW does not completely gap out the Fermi surface. Furthermore, we show that the overall nature of the resistivity does not match the thermally activated transport or the known localization models and warrant further theoretical insight. While the quenched disorder might be an important aspect, to the best of our knowledge, it does not generate similar characteristics in materials other than manganites. Therefore, the interplay of topological states, CDW, magnetism, and lattice disorder must have a distinctive role in the observed properties. We hope that our results would encourage future investigations to confirm this. In addition, the CMR and the giant AHE already make CeSb_0.11_Te_1.90_ an attractive prospect for technological applications.

## MATERIALS AND METHODS

### Single-crystal growth and characterization

Single crystals of CeSb_0.11_Te_1.90_ were grown by chemical vapor transport using iodine as the transport agent. The obtained crystals were characterized by powder/single-crystal x-ray diffraction measurements, and the chemical compositions were determined by energy-dispersive x-ray spectroscopy. Additional details about the crystal growth and characterization can be found in our earlier report (*48*).

### ARPES measurements

ARPES experiments were performed on in situ cleaved crystals in ultrahigh vacuum (pressure below 10^−10^ mbar). The spectra were recorded using the one-cube ARPES setup installed at the UE112-PGM2b beamline at the BESSY-II synchrotron, with various photon energies (*hν*) ranging from 40 to 130 eV.

### Theoretical calculations

Density functional theory calculations were performed in VASP v5.4.4 (*72*–*74*) using the Perdew-Burke-Ernzerhof functional (*75*). Similar to CeSbTe (*40*), localization of the Ce *f*-orbitals was corrected using a Hubbard potential of *U* = 6 eV (*76*). Projector augmented wave potentials (*77*, *78*) were selected on the basis of the v5.4.4 recommendations. Calculation for CeSb_0.11_Te_1.90_ was performed on the 3 × 3 × 2 supercell of Ce_36_Te_68_Sb_4_ with Fermi levels adjusted on the basis of the electron counts obtained from the experimental data. A plane wave energy cutoff of 400 eV and a *k*-mesh density, *l* = 30 (corresponding to 2 × 2 × 2 Γ-centered *k*-meshes for CeSb_0.11_Te_1.90_ supercell), was used. Unfolded spectral functions for the supercells in the subcell BZ were calculated following the method of Popescu and Zunger (*79*) in VaspBandUnfolding.

### Transport measurements

The electronic transport experiments were performed in a physical property measurement system (Quantum Design), equipped with a sample stage rotator using the ac transport option. Prepatterned electrodes in six-probe geometry for resistivity and Hall measurements were deposited directly on the single crystals by gold evaporation. Gold wires were attached to these electrodes using conducting silver paste.
