# Supplementary material for: Colossal magnetoresistance in the multiple wave vector charge density wave regime of an antiferromagnetic Dirac semimetal
Source: Sci Adv. 2023 Oct 13;9(41):eadh0145. doi: 10.1126/sciadv.adh0145 (PMC10575584; doi:10.1126/sciadv.adh0145)
Supplement: Supplementary file 1 — Supplementary Text Figs. S1 to S12 [file sciadv.adh0145_sm.pdf]

Science Advances
AAAS

Supplementary Materials for

Colossal magnetoresistance in the multiple wave vector charge density wave regime of an antiferromagnetic Dirac semimetal

Ratnadwip Singha *et al.*

Corresponding author: Leslie M. Schoop, lschoop@princeton.edu

Sci. Adv. **9**, eadh0145 (2023)
DOI: 10.1126/sciadv.adh0145

This PDF file includes:

Supplementary Text
Figs. S1 to S12

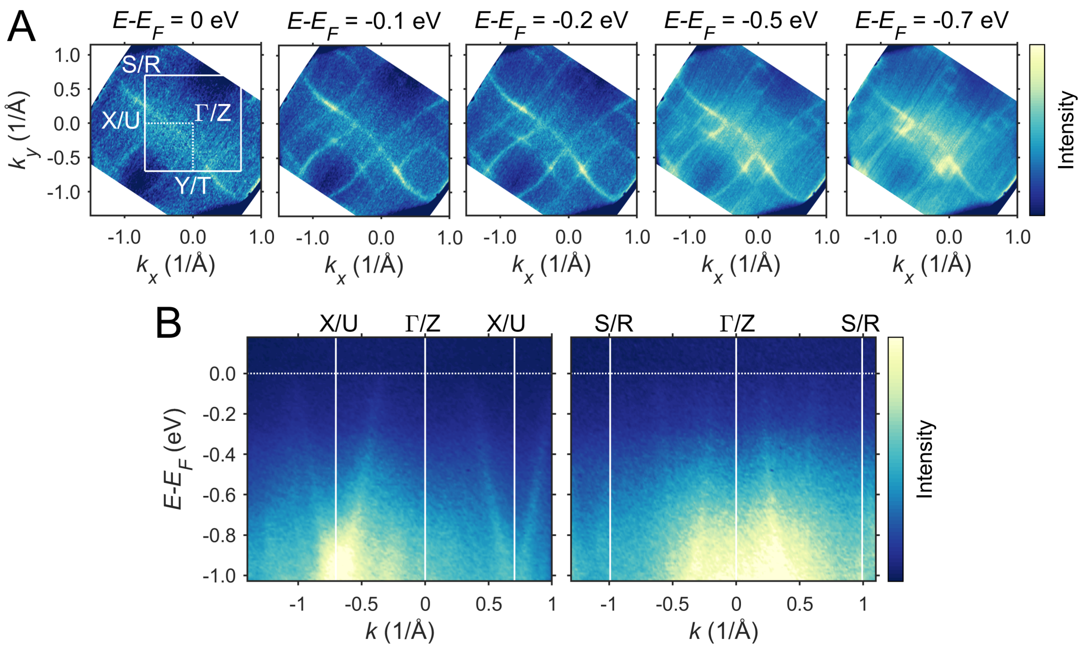

Fig. S 1: **ARPES spectra for $CeSb_{0.11}Te_{1.90}$ at $\sim$30 K measured with a photon energy of 70 eV.** (A) Constant energy contours at different energy (E) values from Fermi energy (E_F) to $E_F-0.7$ eV. (B) Electronic band dispersion along the high symmetry directions $X(U)$-$\Gamma(Z)$-$X(U)$ and $S(R)$-$\Gamma(Z)$-$S(R)$.

Modified symmetrization and anti-symmetrization of the transport data:

The experimentally measured magnetoresistance (MR) and Hall data usually contain components from each other due to unavoidable offset in the placement of the electrodes on the sample. This additional component can be excluded by symmetrization (anti-symmetrization) of the MR (Hall) data over positive (pointing up) and negative (pointing down) magnetic field values. For magnetic sample, however, this method can generate spurious signals as there can be hysteresis between field sweep-up (from negative to positive) and sweep-down (from positive to negative) curves. For example, in $CeSb_{0.11}Te_{1.90}$, the measured Hall resistivity (ρ_{yx}^{raw}) at 2 K in Fig. S11A, demonstrate clear hysteresis between these two curves. To tackle this problem, we measured the resistivity (ρ_{xx}^{raw}) and Hall resistivity for both field sweep up ($H_\uparrow$) and sweep down ($H_\downarrow$) at each temperature. The actual ρ_{xx} and ρ_{yx} are then calculated using the following equations [55],

$$\rho_{xx}(H) = \frac{1}{2}[\rho_{xx}^{raw}(H_\uparrow) + \rho_{xx}^{raw}(-H_\downarrow)] \tag{1}$$

$$\rho_{xx}(-H) = \frac{1}{2}[\rho_{xx}^{raw}(-H_\downarrow) + \rho_{xx}^{raw}(H_\uparrow)] \tag{2}$$

$$\rho_{yx}(H) = \frac{1}{2}[\rho_{yx}^{raw}(H_\uparrow) - \rho_{yx}^{raw}(-H_\downarrow)] \tag{3}$$

$$\rho_{yx}(-H) = \frac{1}{2}[\rho_{yx}^{raw}(-H_\downarrow) - \rho_{yx}^{raw}(H_\uparrow)] \tag{4}$$

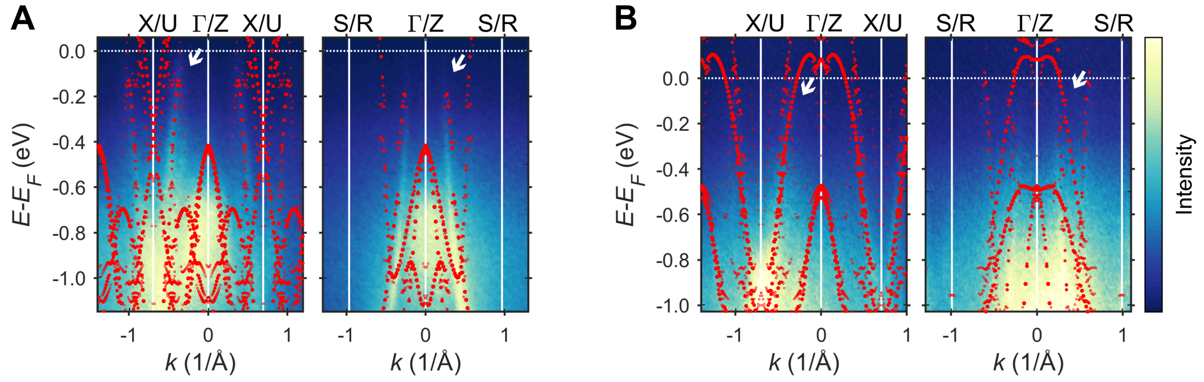

Fig. S 2: **Overlay of the theoretical band structure with the ARPES data** for photon energy (A) 110 eV and (B) 70 eV. The calculated band structure is plotted with red circles along the k-path U-Z-U and R-Z-R in (A) and X-Γ-X and S-Γ-S in (B). Calculated points with weightage less than 4 % of the maximum of the path were excluded from the plot. The arrows highlight the linearly dispersing bands.

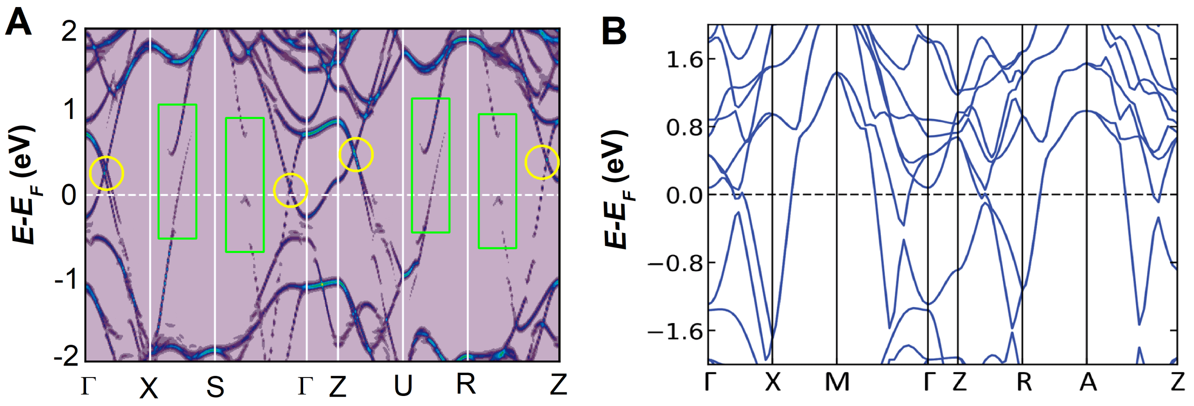

Fig. S 3: **Calculated electronic band structure of $CeSb_{0.11}Te_{1.90}$ over an extended energy range** using (A) the CDW-modulated and (B) a hypothetical unmodulated crystal structure. In panel (A), the positions of the Dirac nodes are highlighted with the yellow circles, whereas the green rectangles show the band modifications by the CDW. The numerical value of the Fermi energy for the hypothetical band structure in panel (B) should not be compared with panel (A).

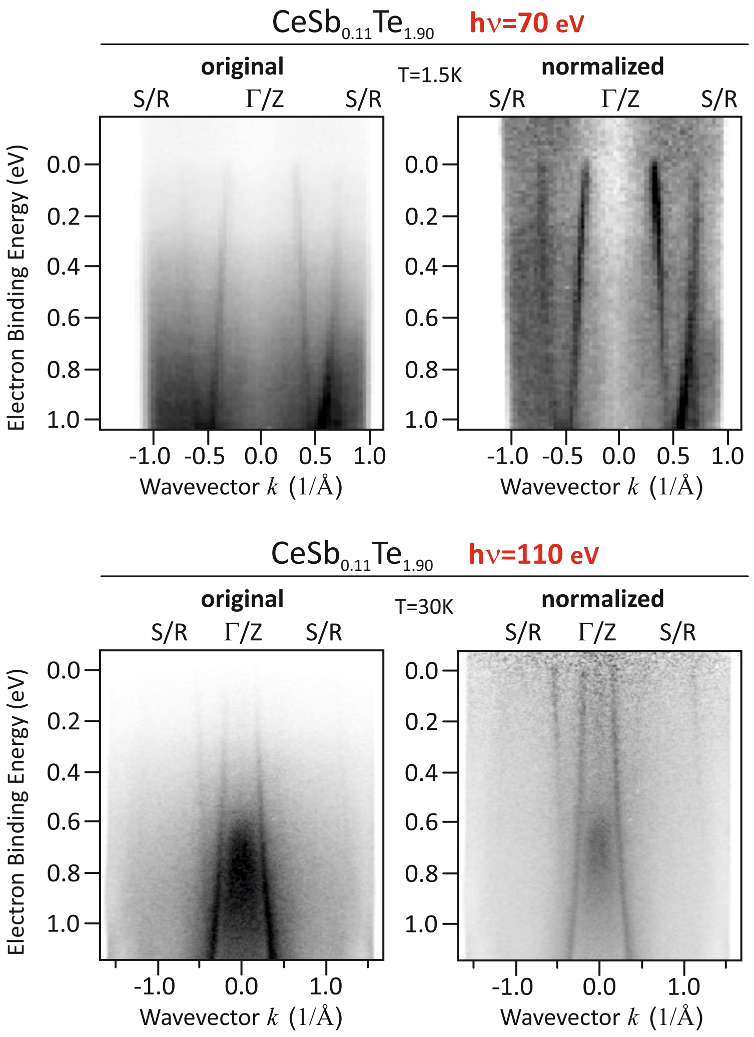

Fig. S 4: ARPES spectrum obtained from the energy distribution curves (EDCs) normalized by the sum of EDCs over all k_x and k_y values for two different photon energies. This method enhances the weak features in the spectrum.

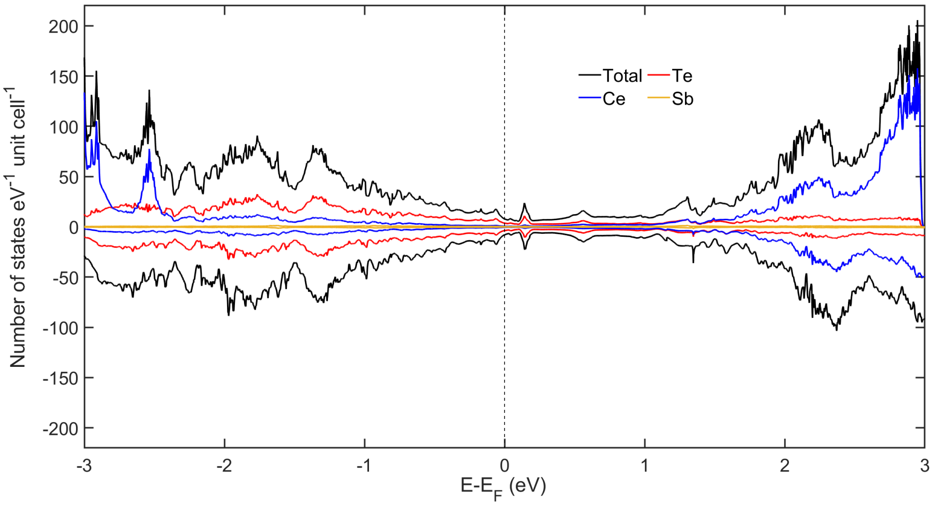

Fig. S 5: **Calculated density of states (DOS) for $CeSb_{0.11}Te_{1.90}$.**

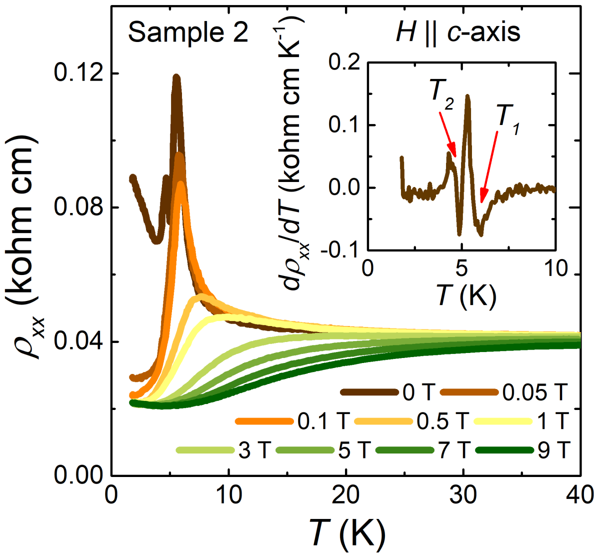

Fig. S 6: **Temperature dependent electronic transport properties for the second crystal (sample 2) from the same batch.** Temperature (T) dependence of the resistivity (ρ_{xx}) at different external magnetic fields applied along the c-axis. The first order derivative of $\rho_{xx}(T)$ is plotted in the inset showing the transition temperatures (vertical dashed lines).

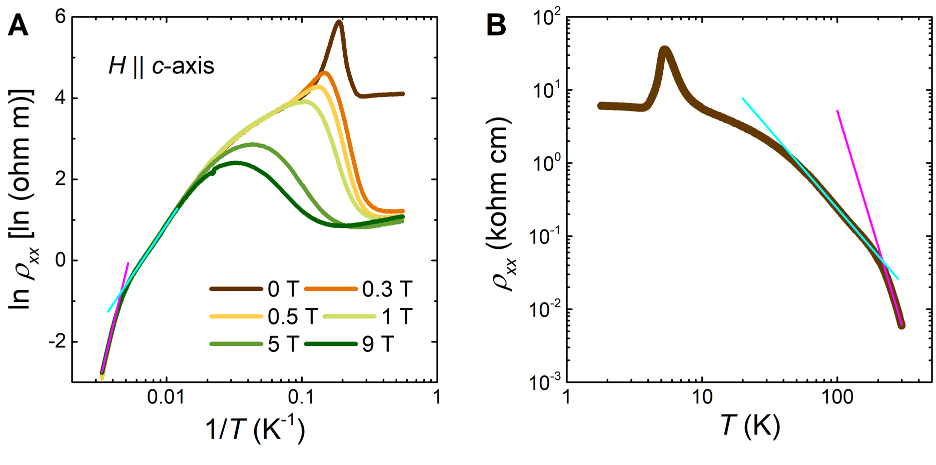

Fig. S 7: **Fitting of the temperature dependent resistivity for $CeSb_{0.11}Te_{1.90}$.** (A) Natural logarithmic resistivity as a function of inverse temperature. The magenta and cyan lines represent fitting of two approximately linear regions. (B) Power-law temperature dependence of the resistivity. The linear fittings (magenta and cyan lines) illustrate two different exponents.

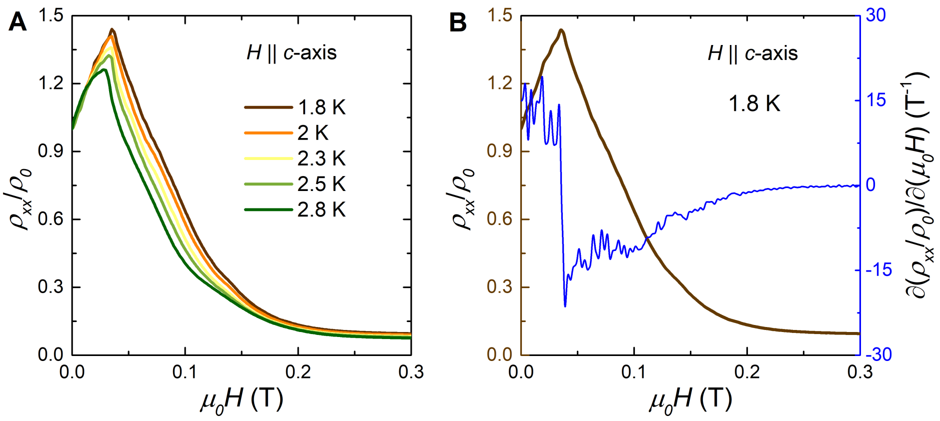

Fig. S 8: **Low magnetic-field region of the normalized resistivity for $CeSb_{0.11}Te_{1.90}$.** (A) Low magnetic-field region of the normalized resistivity (ρ_{xx}/ρ_0) at different temperatures with the field applied along the *c*-axis. A series of transitions can be readily identified. (B) The first order derivative of ρ_{xx}/ρ_0 at a representative temperature 1.8 K to clearly resolve the weak transitions.

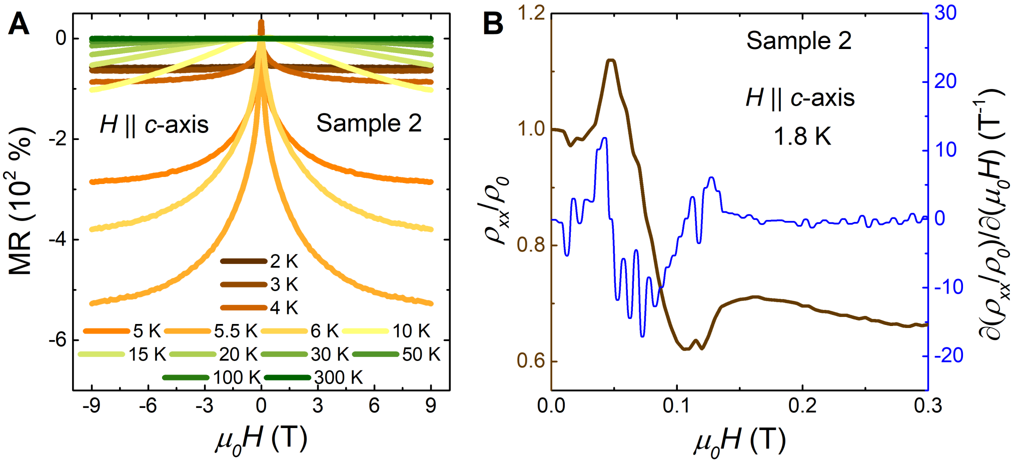

Fig. S 9: **Magnetic field dependent electronic transport properties of sample 2.** (A) Magnetoresistance at different temperatures for magnetic field applied along the *c*-axis. (B) Low-field region of the ρ_{xx}/ρ_0 curve along with the first order derivative with respect to the magnetic field at 1.8 K.

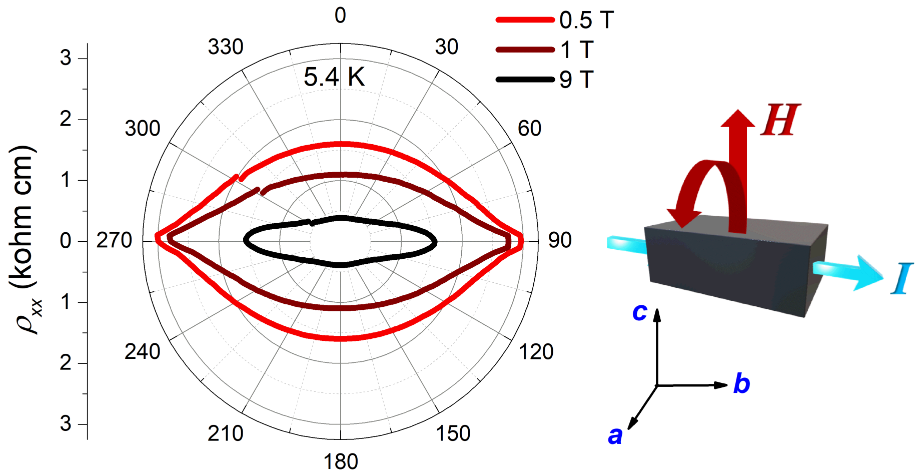

Fig. S 10: **Anisotropy of the magnetic field-dependent resistivity for $CeSb_{0.11}Te_{1.90}$.** Crystallographic direction dependence of the resistivity at 5.4 K with different magnetic field strengths, when the current is along the b-axis and field is rotated in the ac-plane. The schematic illustrates the measurement configuration.

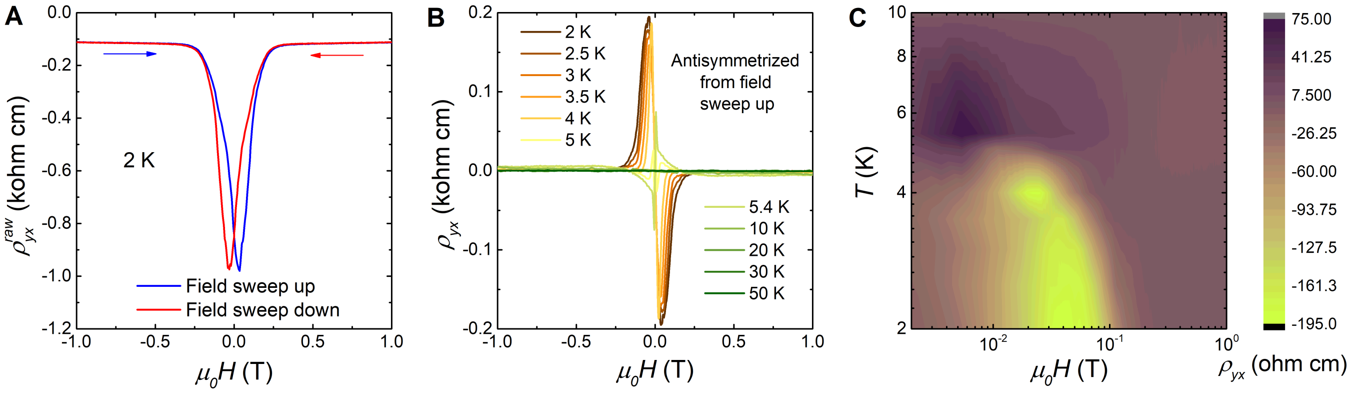

Fig. S 11: **Modified anti-symmetrization of the Hall data for antiferromagnetic $CeSb_{0.11}Te_{1.90}$.** (A) Hysteresis between the magnetic field sweep-up (-9 to 9 T) and sweep-down (9 to -9 T) curves of the raw Hall resistivity (ρ_{yx}^{raw}) data. (B) Extracted Hall resistivity (ρ_{yx}) at different temperatures, obtained by anti-symmetrizing the raw data using a single field sweep. (C) Phase diagram of ρ_{yx}, calculated from a single field sweep.

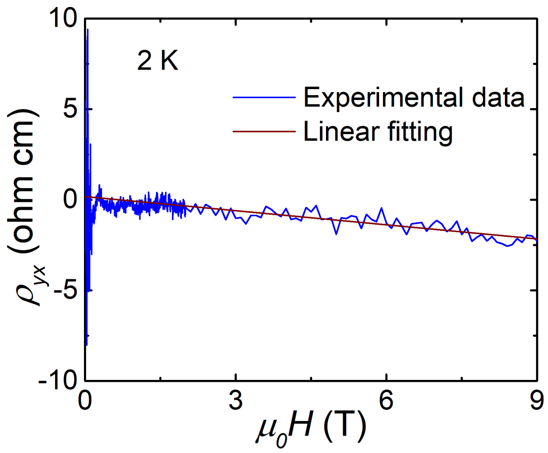

Fig. S 12: **Extraction of the normal Hall component for $\mathbf{CeSb_{0.11}Te_{1.90}}$.** Linear fitting of ρ_{yx} in the high field-region. The extrapolated fitting over the entire field range represents the normal Hall component.
